# Extreme mobility of the world’s largest flying mammals creates key challenges for management and conservation

**DOI:** 10.1186/s12915-020-00829-w

**Published:** 2020-08-21

**Authors:** Justin A. Welbergen, Jessica Meade, Hume E. Field, Daniel Edson, Lee McMichael, Luke P. Shoo, Jenny Praszczalek, Craig Smith, John M. Martin

**Affiliations:** 1 Hawkesbury Institute for the Environment, Western Sydney University, Richmond, NSW 2753 Australia; 2 Department of Agriculture and Fisheries, Queensland Centre for Emerging Infectious Diseases, Brisbane, QLD 4001 Australia; 3 Ecohealth Alliance, New York, NY 10001 USA; 4 School of Veterinary Science, The University of Queensland, Gatton, QLD 4343 Australia; 5 Department of Agriculture, Water and the Environment, Canberra, ACT 2601 Australia; 6 School of Biological Sciences, The University of Queensland, St Lucia, QLD 4072 Australia; 7 Royal Botanic Gardens and Domain Trust, Sydney, NSW 2000 Australia; 8 Institute for Science and Learning, Taronga Conservation Society Australia, Mosman, NSW 2088 Australia

**Keywords:** Bats, Ecosystem services, Human-wildlife conflict, Movement ecology, Nomadic, Zoonosis

## Abstract

**Background:**

Effective conservation management of highly mobile species depends upon detailed knowledge of movements of individuals across their range; yet, data are rarely available at appropriate spatiotemporal scales. Flying-foxes (*Pteropus* spp.) are large bats that forage by night on floral resources and rest by day in arboreal roosts that may contain colonies of many thousands of individuals. They are the largest mammals capable of powered flight, and are highly mobile, which makes them key seed and pollen dispersers in forest ecosystems. However, their mobility also facilitates transmission of zoonotic diseases and brings them in conflict with humans, and so they require a precarious balancing of conservation and management concerns throughout their Old World range. Here, we analyze the Australia-wide movements of 201 satellite-tracked individuals, providing unprecedented detail on the inter-roost movements of three flying-fox species: *Pteropus alecto*, *P*. *poliocephalus*, and *P*. *scapulatus* across jurisdictions over up to 5 years.

**Results:**

Individuals were estimated to travel long distances annually among a network of 755 roosts (*P*. *alecto*, 1427–1887 km; *P*. *poliocephalus*, 2268–2564 km; and *P*. *scapulatus*, 3782–6073 km), but with little uniformity among their directions of travel. This indicates that flying-fox populations are composed of extremely mobile individuals that move nomadically and at species-specific rates. Individuals of all three species exhibited very low fidelity to roosts locally, resulting in very high estimated daily colony turnover rates (*P*. *alecto*, 11.9 ± 1.3%; *P*. *poliocephalus*, 17.5 ± 1.3%; and *P*. *scapulatus*, 36.4 ± 6.5%). This indicates that flying-fox roosts form nodes in a vast continental network of highly dynamic “staging posts” through which extremely mobile individuals travel far and wide across their species ranges.

**Conclusions:**

The extreme inter-roost mobility reported here demonstrates the extent of the ecological linkages that nomadic flying-foxes provide across Australia’s contemporary fragmented landscape, with profound implications for the ecosystem services and zoonotic dynamics of flying-fox populations. In addition, the extreme mobility means that impacts from local management actions can readily reverberate across jurisdictions throughout the species ranges; therefore, local management actions need to be assessed with reference to actions elsewhere and hence require national coordination. These findings underscore the need for sound understanding of animal movement dynamics to support evidence-based, transboundary conservation and management policy, tailored to the unique movement ecologies of species.

## Background

Conventional conservation approaches, which typically view species as organized around discrete local populations, are inadequate for highly mobile species [1], particularly in the context of environmental change [2]. Highly mobile species often require multiple habitats to obtain different resources at different stages of their life cycles, and their persistence depends on the availability and accessibility of the requisite suite of habitats [3, 4]. The unpredictable movements of nomadic species make it particularly difficult to decide where and how to act to mitigate threatening processes [5]. This can be further complicated when such species cross jurisdictional boundaries within or between countries [6], making a unified program of conservation management much more difficult to achieve. For effective conservation management, it is essential to have a robust understanding of the movement ecology of highly mobile species, but this can only be accomplished by following numerous individuals within a population, across multiple habitats within the species’ range [7, 8].

Australian flying-foxes (*Pteropus* spp.) are large bats that forage by night on floral resources and rest by day in arboreal roosts that may contain colonies of many thousands of individuals [9] with a complex social architecture [10, 11]. Roost locations can be stable for decades [12], and while “traditional” sites are mostly occupied seasonally, more recent, urban roosts are occupied permanently [13], albeit with great seasonal variation in local numbers [14]. The prevailing assumption is that flying-foxes are organized around local “resident” populations that show (seasonal) fidelity to a particular site [13]. However, like other large pteropodids elsewhere (e.g., [15–22]), Australian flying-fox individuals can be highly mobile, with movements ranging from small relocations within roosts and foraging sites [10] to nightly foraging trips of up to 80 km [23, 24] and long-distance movements of several thousand kilometers [25, 26]. Therefore, how flying-fox populations are locally organized is critically dependent on the extent and seasonal dynamics of movements among roosts. To date, as for the other large pteropodids elsewhere (e.g., [15–22]), movement studies of Australian flying-foxes are limited to small samples of radio- [23, 27–29] and satellite-tracked [21, 25, 26] individuals, so the extent and seasonal dynamics of movements among roosts have never been formally assessed, hampering effective conservation and management of these ecologically important species.

The mobility of flying-foxes is thought to enable them to exploit Australia’s ephemeral floral resources [30] and makes them key long-distance pollen and seed dispersers [31–33]. Long-distance seed and pollen dispersal by all four Australian mainland *Pteropus* species (*Pteropus alecto*, *P*. *poliocephalus*, *P*. *scapulatus*, and *P*. *conspicillatus*) would be of crucial conservation significance as it promotes gene flow between impoverished forest patches and facilitates range shifts of forage trees under climate change [34, 35]. Knowledge on the extent and seasonal dynamics of movements among roosts is thus key for understanding the linkages that flying-foxes provide in Australia’s contemporary fragmented landscape.

The mobility of flying-foxes is also thought to underpin their role in the ecology of several emerging infectious diseases. In Australia, flying-foxes are the recognized natural hosts for various viral agents that threaten livestock and/or human health, including Australian bat lyssavirus (ABLV) [36], Hendra virus [37, 38], and Menangle virus [39]. The maintenance of infection in natural host populations depends on a source of infection, a continuous supply of susceptible individuals, and adequate contact between infected and susceptible individuals. Thus, the extent and seasonal dynamics of flying-fox movements are expected to shape infection and transmission dynamics at the roost and metapopulation level; further, they define the spatiotemporal scales of exposure and infection potential for susceptible livestock species and humans [40].

The mobility of flying-foxes further puts them in frequent conflict with humans. Over the last 20 years, Australian flying-foxes have increasingly exploited urban foraging and roosting resources [23, 41, 42]. Many urban areas in eastern Australia now have permanent flying-fox colonies [13], and this increased urban presence translates to increased interaction with humans, and can provoke negative community sentiment due to objectionable noise, soiling and smell, and impacts on human health [43–45]. The result is often public demands to local councils and elected members of state and federal electorates for aggressive management of urban flying-fox populations, ranging from roost vegetation modification to colony dispersal. Dispersals in particular are predicated on the notion that resident individuals can learn to avoid locations where they are not wanted; however, if colonies are in fact composed of highly mobile individuals that turnover at high rates, this could explain why dispersal actions are commonly met with very limited success [46].

In summary, despite their key importance for Australia’s fragmented forest ecosystems, flying-foxes are contentious in terms of zoonosis and human-wildlife conflict and so require a precarious balancing of conservation, animal welfare, and human health and amenity concerns. However, the conservation and management of flying-foxes is complicated by their trans-jurisdictional distributions and by conventional notions that they are organized around discrete local populations (colonies). A comprehensive understanding of the extent and seasonal dynamics of flying-fox movements is thus vital for effective trans-jurisdictional conservation and management of the species.

In this study, we capitalize on recent advances in satellite tracking technology to investigate the broad-scale inter-roost movement patterns of an unprecedented 201 flying-foxes in eastern Australia. We describe in detail the nature of the continental-scale movements of *P*. *alecto*, *P*. *poliocephalus*, and *P*. *scapulatus* and the differences between these species in terms of local site fidelity and the spatiotemporal extents of their movements among roosts and local jurisdictions. We discuss the implications of our findings for the ecosystem services and zoonotic dynamics of flying-fox populations and for current practices in flying-fox conservation and management.

## Results

A total of 201 transmitters was deployed on 80 *P*. *alecto*, 109 *P*. *poliocephalus*, and 12 *P*. *scapulatus*, and tagged individuals were tracked over a maximum period of 60 months (Additional file 1: Table S1; see Additional file 2: Video S1 for the animated movements of all 201 tracked individuals, and for each species separately (Additional file 3: Video S2, Additional file 4: Video S3, Additional file 5: Video S4)).


**Additional file 2: Video S1.** Movements of all satellite-tracked (*n* = 201) individuals, color-coded by species. Straight-line movements between recorded fixes are interpolated. The box in the top right shows the month and the year; whereas the box in the top left shows the number of individuals being tracked concurrently for each species.**Additional file 3: Video S2.** Movements of all satellite-tracked *P*. *alecto* (*n* = 80) individuals only.**Additional file 4: Video S3.** Movements of all satellite-tracked *P*. *poliocephalus* (*n* = 109) individuals only.**Additional file 5: Video S4.** Movements of all satellite-tracked *P*. *scapulatus* (*n* = 12) individuals only.

### Roost sites

**Fig. 1:**
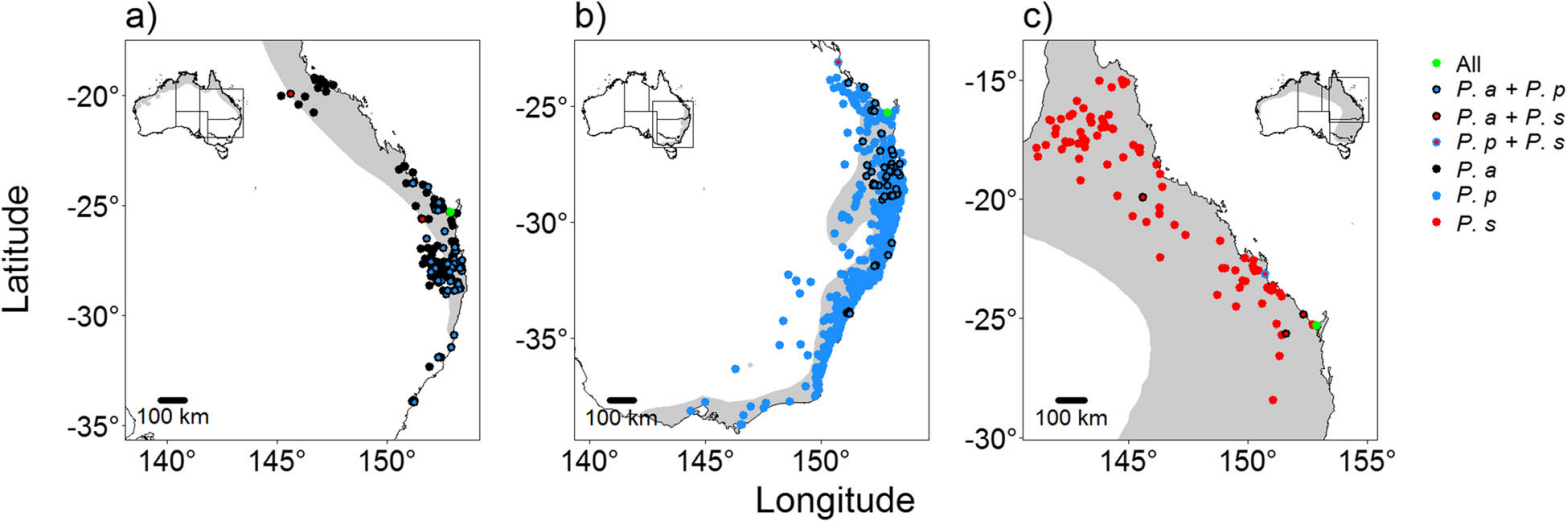
Daytime roost sites used by satellite-tracked individuals. **a** *Pteropus alecto*. **b** *P*. *poliocephalus*. **c** *P*. *scapulatus*. Dots are colored to indicate which species of tracked animal used the roost sites. See legend for more details. Insets: Maps with shaded areas indicating the IUCN species range in Australia; lines indicate state boundaries

Following release from eight colonies, tracked flying-foxes used a total of 755 roost sites, of which 458 (61%) were previously unrecorded. Of these new sites, 123 (26%) were used by multiple tracked individuals and we thus considered them to accommodate previously unidentified flying-fox “colonies” (see the “Methods” section). Roost sites spanned a north-south distance of 2698 km (23.7 degrees of latitude) and an east-west distance of 1099 km. *P*. *alecto* was identified roosting at 173 sites, *P*. *poliocephalus* at 546 sites, and *P*. *scapulatus* at 89 sites. One roost site (Hervey Bay Botanic Gardens) was used by tracked individuals of all three species; 47 roost sites were used by only *P*. *alecto* and *P*. *poliocephalus*, one roost site was used by only *P*. *poliocephalus* and *P*. *scapulatus*, and three roost sites were used by only *P*. *alecto* and *P*. *scapulatus* (Fig. 1).

### Jurisdictions

**Fig. 2:**
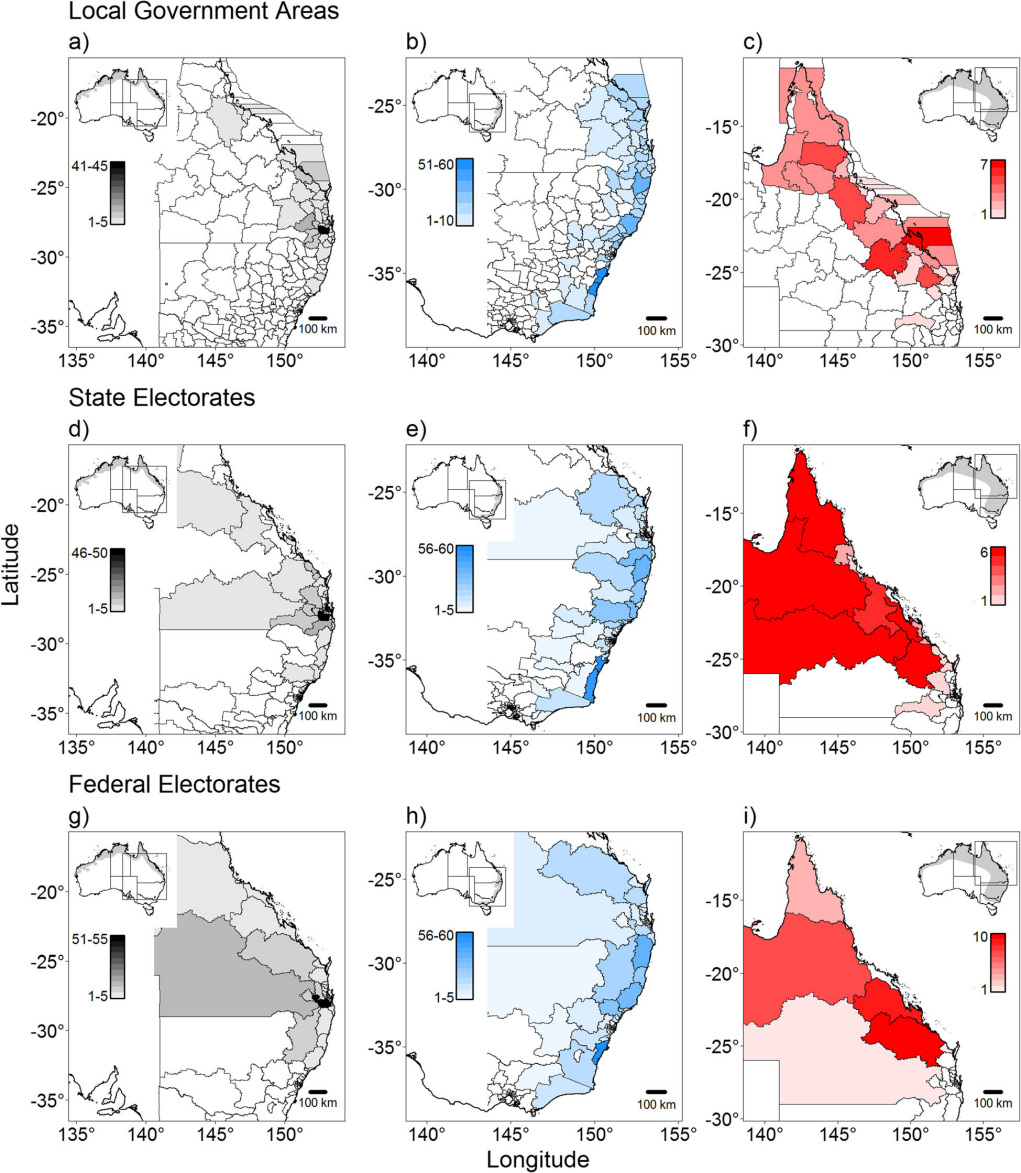
The numbers of satellite-tracked individuals found within Australian jurisdictions. **a**–**c** Local government areas. **d**–**f** State electorates. **g**–**i** Federal electorates. Colors denote species: black: *Pteropus alecto*; blue: *P*. *poliocephalus*; red: *P*. *scapulatus*. Insets: Maps with shaded areas indicating the IUCN species range in Australia; lines indicate state boundaries

Tracked flying-foxes roosted in a total of 101 local government areas (LGAs; also known as “councils”) within 131 state electorates and 74 federal electorates. *P*. *alecto* individuals roosted in a total of 36 LGAs (average 12.2 year^−1^, range 1–9) within 57 (average 13.2 year^−1^, range 1–9) state electorates and 33 (average 12.0 year^−1^, range 1–8) federal electorates; *P*. *poliocephalus* individuals roosted in a total of 85 LGAs (average 8.1 year^−1^, range 1–37) within 109 (average 8.2 year^−1^, range 1–32) state electorates and 68 (average 6.7 year^−1^, range 1–24) federal electorates; *P*. *scapulatus* individuals roosted in a total of 21 LGAs (average 23.8 year^−1^, range 1–9) within 16 (average 21.1 year^−1^, range 1–9) state electorates and 6 (average 16.2 year^−1^, range 1–4) federal electorates (Fig. 2).

### Movements among roost sites

**Fig. 3:**
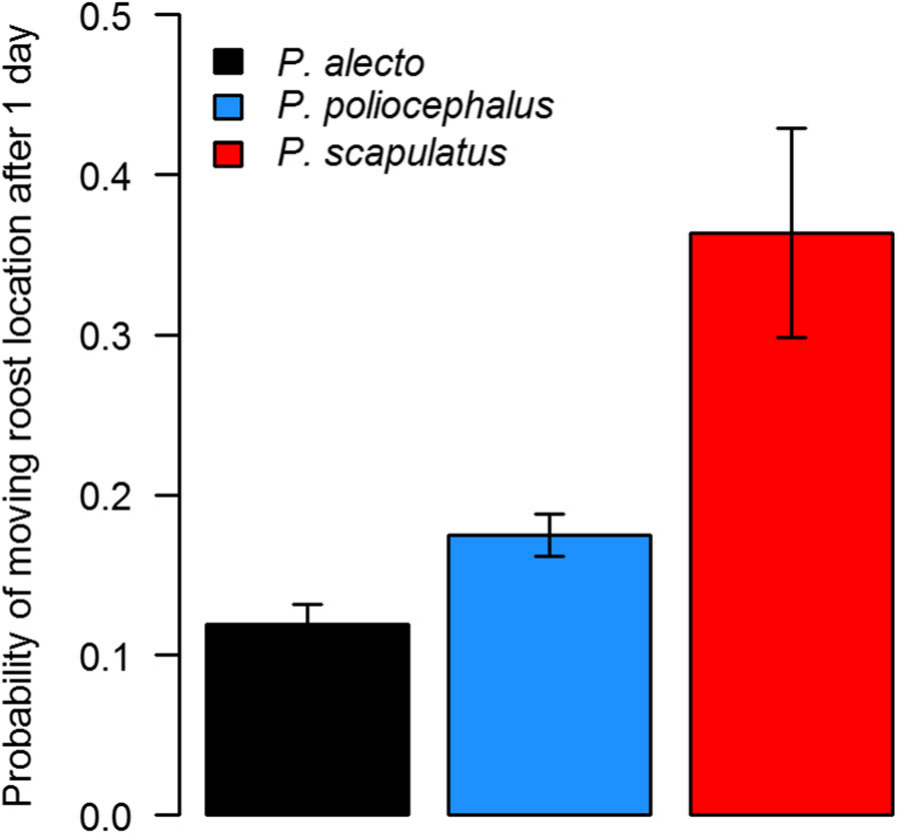
The probability that an individual changes roost location after 1 day (± 1 SE) for each species (this provides an estimate of the average daily colony turnover rate for each species, assuming the behavior of tracked individuals was representative of that of all individuals within the species). There was a significant difference in the probability that an individual changed roost location after 1 day between the species (*P*. *alecto* vs. *P*. *poliocephalus*: *p* = 0.002; *P*. *alecto* vs. *P*. *scapulatus*: *p* < 0.001; *P*. *poliocephalus* vs. *P*. *scapulatus*: *p* < 0.001)

There was a significant difference in site fidelity (i.e., the inverse of the probability of moving between roosts) between the three species (*P*. *alecto* vs. *P*. *poliocephalus*: *p* = 0.002; *P*. *alecto* vs. *P*. *scapulatus*: *p* < 0.001; *P*. *poliocephalus* vs. *P*. *scapulatus*: *p* < 0.001), with the best fitting model including the additive effect of species and days since last daytime fix (Additional file 6: Table S2). *P*. *scapulatus* had the highest daily propensity (and thus the lowest daily site fidelity) for moving between roost sites (0.364 ± 0.065 SE), followed by *P*. *poliocephalus* (0.175 ± 0.013) and *P*. *alecto* (0.119 ± 0.013) (Fig. 3).

### Distances moved between roost sites

The mean estimated distance moved between roost sites was greatest for *P*. *scapulatus* at 13.57 ± 1.79 km day^−1^ SE (range 0–162 km day^−1^), followed by 4.26 ± 0.14 km day^−1^ for *P*. *poliocephalus* (range 0–270 km day^−1^), and 1.68 ± 0.14 km day^−1^ for *P*. *alecto* (range 0–92 km day^−1^) (Additional file 7: Fig. S1), suggesting that the species travel 4956, 1554, and 612 km on average among roost sites annually, respectively.

Nevertheless, some individuals are clearly capable of traveling much greater annual distances among roosts. For example (representing the maximum distances traveled by each species), *P*. *alecto* (#112209) covered 1551 km between 38 roosts (within 2 LGAs, 2 state electorates, and 2 federal electorates) across 289 tracking days (5.36 km day^−1^, and could be scaled up to 1959 km year^−1^); *P*. *poliocephalus* (#114099) covered 12,337 km between 123 roosts (within 37 LGAs, 30 state electorates, and 21 federal electorates) across 1629 tracking days (7.57 km day^−1^; 2764 km year^−1^); and *P*. *scapulatus* (#112212) covered 3255 km between 36 roosts (within 9 LGAs, 9 state electorates, and 4 federal electorates) across 194 tracking days (16.78 km day^−1^, and could be scaled up to 6124 km year^−1^).

In reality, flying-foxes likely traveled much greater distances between roosts than the straight-line distances inferred from tracking data suggest, because fixes were only obtained once every 3–10 days and any roosts visited on these “off days” were missed. To account for such missed intervening roost visits, we modeled the expected daily distances moved between roost sites by taking advantage of the variation in the time elapsed between fixes (see the “Methods” section). From this, we derived daily inter-roost movement distances of 13.50 ± 3.138 km (x̅ ± 95% CI) for *P*. *scapulatus* (= 311–499 km/month; 3782–6073 km year^−1^), 6.62 ± 0.405 km day^−1^ for *P*. *poliocephalus* (= 186–211 km/month; 2268–2564 km year^−1^), and 4.54 ± 0.630 km day^−1^ for *P*. *alecto* (= 117–155 km/month; 1427–1887 km year^−1^) (Additional file 8: Fig. S2).

**Fig. 4:**
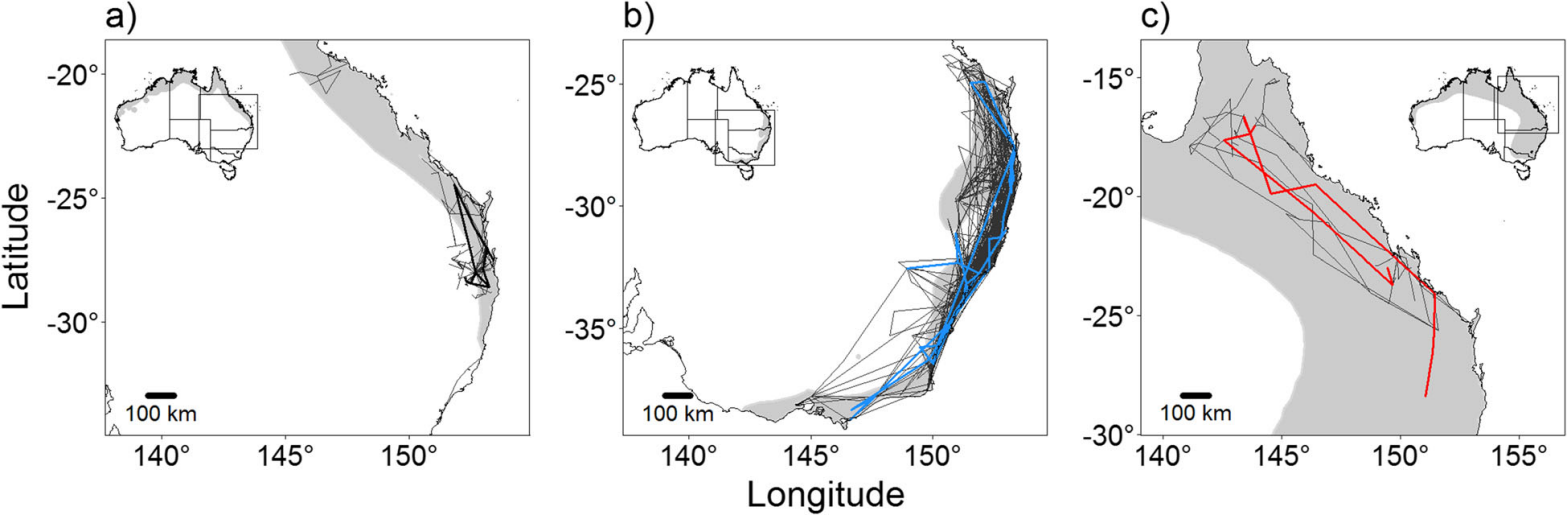
Straight-line connections between successive roost fixes of satellite-tracked individuals. **a** *Pteropus alecto*. **b** *P*. *poliocephalus*. **c** *P*. *scapulatus*. Paths highlighted by thick lines indicate the tracks of the single individual of each species covering the greatest latitudinal range: black *Pteropus alecto* individual (#117723), tracked for 7 months from 25 June 2013 to 12 March 2014; blue *P*. *poliocephalus* individual (#114111), tracked for 21 months from 11 May 2012 to 12 November 2014; and red *P*. *scapulatus* individual (#112212), tracked for 6.5 months from 03 May 2012 to 16 November 2012. Insets: Maps with shaded areas indicating the IUCN species range in Australia; lines indicate state boundaries

While much of the travel distances represent movements among nearby roosts, some individuals covered extensive latitudinal distances, (repeatedly) traversing substantial proportions of their entire species range. For example, one *P*. *alecto* individual (#117723) covered 4.13 degrees of latitude between 23 roosts (within 8 LGAs, 6 state electorates, and 7 federal electorates) across 260 tracking days (Fig. 4a); one *P*. *poliocephalus* individual (#114111) covered 13.78 degrees of latitude between 182 roosts (within 25 LGAs, 24 state electorates, and 17 federal electorates) across 2093 tracking days (Fig. 4b); and one *P*. *scapulatus* individual (#112212) covered 11.77 degrees of latitude between 36 roosts (within 9 LGAs, 9 state electorates, and 4 federal electorates) across 197 tracking days (Fig. 4c).

### Directional movements

Evidence of concerted directional movements of animals of each species was mixed. When monthly directional movements among roosts were examined within species, we found that *P*. *alecto* individuals were significantly oriented (in a single direction) in 1 of 10 months; *P*. *poliocephalus* were significantly oriented in 19 of 41 months, with a single preferred direction occurring in 9 of those months. *P*. *scapulatus* were significantly oriented (in a single direction) in both of the months where the sample size exceeded 5 (Additional file 9: Fig. S3).

Despite the lack of uniformity of monthly inter-roost movement directions (see above), *P*. *poliocephalus* exhibited a significant seasonal north-south signal in their movements overall (dev = 47.1; df = 12, *P* < 0.001). No significant seasonal movement was detected for *P*. *alecto* (dev = 0; df = 0, *P* = 1). As data for *P*. *scapulatus* were limited to a single year, no test for seasonality could be performed; however, like *P*. *poliocephalus*, *P*. *scapulatus* tended to spend more time further north on average in winter than in summer (Additional file 10: Fig. S4).

## Discussion

Fundamentally, movement creates challenges for the conservation and management of species, in part because animal movements may transcend the jurisdictional boundaries of single agencies or countries [47, 48]. The extreme mobility of flying-foxes vividly illustrates these challenges and highlights the need for a sound understanding of the mechanisms underpinning movement dynamics to support evidence-based wildlife management policy and infectious disease risk mitigation [49]. Further, we identified a clear spatiotemporal component of movement, roost occupancy and by extension, resource utilization, requiring conservation management and potential disease risk mitigation to be tailored to the unique movement ecology of each species.

The scale and scope of our study provides unprecedented detail on the mobility of *P*. *alecto*, *P*. *poliocephalus*, and *P*. *scapulatus* in eastern Australia over more than 23 degrees of latitude and up to 5 years. These findings extend those of previous, smaller studies [21, 25, 26] by demonstrating that flying-foxes undertake frequent inter-roost movements at a regional level as well as longer-range, and at times seasonal, movements. The annual inter-roost distances reported in our study rank all three *Pteropus* among the most mobile mammals on earth, above large-bodied ungulates and most cetaceans, and in the same range as migratory birds [50], despite our results necessarily underestimating flying-fox movement distances.

Our findings further show that the three *Pteropus* species are composed of highly dynamic populations of individuals moving among roosts in different directions, at different rates (see Electronic SI 1–4). This extreme inter-roost mobility is consistent with genetic work that shows that the species are panmictic across their ranges [51, 52], and has important implications for the ecosystem services and zoonotic dynamics of flying-fox populations and for current management practices in flying-fox conservation and human-wildlife conflict mitigation.

### Implications for the role of flying-foxes in Australia’s fragmented landscape

Flying-foxes are thought to be pivotal to forest ecosystems as pollinators and seed dispersers [31, 53], providing linkages between habitat fragments across anthropogenic [32] and natural barriers [21, 54]. Australia has lost approximately 38% of native forests since European settlement [33], and the number and geographic span of roosts identified in this study, together with the scale of movement among them, graphically illustrates the extent of the linkages that flying-foxes provide in Australia’s contemporary fragmented landscape.

In Australia, the spatiotemporal distribution of resources is often unpredictable and animals must either be generalists and survive scarcity without relocating or be highly mobile and track resource availability across large spatial scales [30]. Our finding of no (*P*. *alecto*) or weakly (*P*. *poliocephalus* and *P*. *scapulatus*) concerted monthly movement directions suggests that at least at these large spatial scales flying-foxes do not track resources using environmental cues or memory; rather, individuals appear to move in a quasi-random, or Lévy flight-like fashion, which is thought to be optimal for searching sparsely and randomly distributed targets in the absence of memory [55]. In this view, individuals wander freely across the species range but slowdown in more attractive or “sticky” areas where foraging resources are temporarily plentiful. Here, they combine with other individuals that encounter the resources from elsewhere, and when local resources are depleted, individuals again diffuse nomadically across the range. While largely speculative at this stage, this scenario could account for the local build-up of individuals during mass flowering events [56] and for the recent increase in the stability of urban roosts [41], phenomena for which the mechanisms are currently unexplained (but see, [57]).

### Implications for infection and transmission dynamics of zoonotic agents

The differential movement behavior among species is important for better understanding Hendra virus infection and transmission dynamics, and spillover risk. Hendra virus, associated with around 100 fatal equine cases [58] and four fatal human cases in QLD and NSW, appears to be primarily excreted by *P*. *alecto* and *P*. *conspicillatus* [59–62]. Virus excretion has not been detected in *P*. *poliocephalus* or *P*. *scapulatus* to date, although anti-Hendra virus antibodies have been reported in both species [62, 63]. One explanation for this is that infection is not maintained in *P*. *poliocephalus* or *P*. *scapulatus*, but that they are periodically exposed to the virus. Urine is the primary route of Hendra virus excretion in *P*. *alecto* [43, 64], and co-roosting *P*. *poliocephalus* or *P*. *scapulatus* will have repeated exposure to *P*. *alecto* urine. Thus, our findings of extensive movements by *P*. *poliocephalus* and *P*. *scapulatus* and the co-roosting of both with *P*. *alecto* suggest a mechanism for interspecies viral exposure. Further, given the lack of evident Hendra virus excretion in *P*. *poliocephalus* and *P*. *scapulatus*, our findings illustrate the potential for high risk roosts (in terms of virus excretion and equine exposure risk) where *P*. *alecto* are present and low risk roosts where only *P*. *scapulatus* or *P*. *poliocephalus* are present (Fig. 1). However, such roost risk profiles are not static and are likely determined by roost species composition and modulated by geographic location or latitudinal factors. Indeed, the reported southern range expansion of *P*. *alecto* [65] suggests the likelihood of higher Hendra virus risk roosts further south in coming years.

Roost fidelity of *P*. *alecto* was relatively higher compared to the other species, which initially appears inconsistent with its Hendra virus reservoir role; however, *P*. *alecto* colonies were still expected to turnover at approximately 12% per day (Fig. 3), providing enormous potential for transmission between roosts. Thus, the potential for infection to disseminate across the geographic range of the species is clear and underscored by the geographic occurrence of equine cases [58].

### Implications for conservation management

We found that roosting at unknown sites was common (458 out of a total of 755 sites used), and we identified 123 previously unknown sites that hosted multiple tracked individuals (and so were classified as “colonies” by our definition). Currently, changes in the abundance and distribution of *P*. *alecto*, *P*. *poliocephalus*, and *P*. *scapulatus* are estimated through Australia’s National Flying-Fox Monitoring Program [66], and roosting away from known roosts is identified as the major contributor to uncertainty around flying-fox population trend estimates [67, 68]. We suggest that the accuracy of the monitoring could thus be substantially improved by the annual inclusion of tracked individuals to help reveal previously unidentified roosts.

Our findings have particular relevance for the conservation management of *P*. *poliocephalus* as this species used 30% of new roosts and 70% of all roosts. *P*. *poliocephalus* is classified as “vulnerable to extinction” in The Action Plan for Australian Bats [69] and listed as “vulnerable” on the IUCN Red List of Threatened Species. Threats include loss of foraging habitat [70], extreme temperature events [71], and human persecution [41]. None of these threats have abated and have recently been compounded by the unprecedented bush fires during 2019–2020 that burnt an estimated 5.8 Mha of temperate broadleaf forest within *P*. *poliocephalus*’ range [72]. It is clear from the vast spatial extent of inter-roost movements reported here (e.g., Fig. 4b) that successful conservation management of *P*. *poliocephalus* (and other flying-foxes) must be enacted across the entire species range.

### Implications for human-wildlife conflict mitigation

Our findings show that a flying-fox colony comprises a highly fluid subset of highly nomadic individuals from across the species range, and the size of a colony at any given time would thus reflect the net outcome of opposing influx and outflux of such mobile individuals. This contrasts with the conventional portrayal of a roost as being inhabited by flying-foxes with a “strong fidelity” to a roost, and our findings require a reappraisal of the concept of a “local population” in a “single locality” that is used, for example, in the assessment of impacts of management actions on the species [73].

Flying-fox roost management actions range from roost vegetation modification to colony dispersal [74, 75], but these actions often inadvertently exacerbate the human-wildlife conflict they aim to resolve [46]. “Dispersal” actions implicitly assume that the individuals that are present at the time of active management are those that are “dispersed.” However, our results indicate that locally, individuals in fact turnover at extremely high rates (Fig. 3). This explains why repeat “maintenance dispersals” are required in the majority of actions [76] because naïve individuals continue to arrive at a site without knowledge of previous dispersal activities. Further, flying-foxes tend to arrive at a roost around dawn and are extremely reluctant to cover great distances during daylight hours possibly due to increased risk of predation [77] and thermophysiological limitations [78]; therefore, they have no choice but to attempt to roost in the nearest available site where they provide a “seed” around which a new “splinter colony” can form. This can explain the local proliferation of human-wildlife conflict that is commonly observed following dispersal actions [76]. It is thus essential that the extreme mobility of flying-foxes and the highly dynamic nature of their colonies now become integral components of the local management of the species.

In Australia, flying-fox management actions are currently implemented locally at the level of councils without adequate coordination at both state and federal levels. Yet, the extreme mobility of tracked flying-foxes among the large number of councils (101; Fig. 2) clearly indicates that local management actions are likely to affect, and complicate, the management of flying-foxes by councils elsewhere. Furthermore, councils often enact dispersals in response to top-down pressure from members from state and federal electorates. Yet, the extreme mobility of tracked flying-foxes among the large number of state (131) and federal electorates (74) (Fig. 2) clearly indicates that such pressure can have negative implications for flying-fox management across other jurisdictions and so is not without political cost. Moreover, current lack of coordinated state and federal oversight means that management actions can be implemented locally by councils without reference to the impacts on the species from management actions elsewhere. Yet, in the case of vulnerable *P*. *poliocephalus*, tracked individuals on average visited 8.1 council areas, and 8.2 state and 6.7 federal electorates per year, clearly demonstrating the high potential for cumulative impacts from local management actions on the conservation of this species.

## Conclusions

Our work shows that a flying-fox roost forms a “node” in a network of “staging posts” through which highly nomadic individuals travel far and wide across their species range, which has profound implications for the ecosystem services and zoonotic dynamics of flying-fox populations. In addition, the extreme inter-roost mobility reported here also means that impacts from local management actions can readily reverberate across jurisdictions; hence, local management actions should be formally assessed in light of the impacts of actions undertaken elsewhere, urgently necessitating more holistic coordination at the national scale. As such, our study provides a warning of how management at inappropriate scales can potentially have unforeseen widespread consequences for population processes and ecological functioning in mobile species.

## Methods

### Capture and transmitter deployment

We deployed transmitters at eight roosts in the Australian states of Queensland (QLD) and New South Wales (NSW) between January 2012 and May 2015, as a component of three discrete studies. In QLD, we caught and released in situ flying-foxes at Boonah (− 28.0° S,152.7° E; *n* = 56 *P*. *alecto*), Charters Towers (− 20.1° S, 146.3° E; *n* = 4 *P*. *alecto*), Duaringa (− 23.7° S, 149.7° E; *n* = 4 *P*. *scapulatus*), Gayndah (− 25.6° S, 151.7° E; *n* = 4 *P*. *alecto*, 8 *P*. *scapulatus*), Loders Creek (− 28.0° S, 153.4° E; *n* = 4 *P*. *alecto*), Parkinson (− 27.6° S, 153.0° E; *n* = 10 *P*. *poliocephalus*) and Toowoomba (− 27.6° S, 151.9° E; *n* = 10 *P*. *alecto*), and in NSW at the Royal Botanic Garden, Sydney (− 33.9° S, 151.2° E; *n* = 2 *P*. *alecto*, 100 *P*. *poliocephalus*).

We caught flying-foxes returning to roost pre-dawn using mist-nets (12–18 m wide and 2.4–4.8 m deep) hoisted between two 15–20 m masts situated adjacent to the target roost. We continuously attended nets and immediately lowered them when a bat became entangled. The bat was physically restrained and placed in an individual cotton bag [79].

The criteria for recruitment for transmitter deployment were health (no evident injury or illness) and body mass (> 550 g for *P*. *alecto* and *P*. *poliocephalus*; > 350 g for *P*. *scapulatus*). The accepted proportion of bodyweight of the device is 5% or less [80], and we aimed to minimize the proportion of bodyweight where possible. In NSW, deployment was limited to *P*. *poliocephalus* individuals ≥ 650 g. We sequentially anesthetized all captured bats using the inhalation agent isoflurane [81] and estimated age (juvenile or adult) from dentition [82] and the presence or absence of secondary sexual characteristics [43, 83, 84]. Bats meeting the criteria were fitted with collar-mounted transmitters immediately prior to recovery from anesthetic. All bats were recovered from anesthesia, offered fruit juice, and released at their capture location within 5 h of capture.

### Platform terminal transmitter specifications, application, and operation

Microwave Telemetry 9.5 g (*n* = 150) and GeoTrak 12 g (*n* = 52) solar platform terminal transmitter (PTT) units were mounted on lightweight flexible collars. The QLD collar was a modified nylon webbing proprietary small dog collar whose overlapping ends were secured with an absorbable suture material, allowing the collar to drop off after an estimated 4–6 months. The NSW collar was neoprene–lined leather whose overlapping ends were secured by a ferrous rivet, providing extended deployment time. The combined transmitter/collar weight was < 20 g, translating to < 3.7% of the minimum recruited body mass for *P*. *alecto* and *P*. *poliocephalus*, and < 5.7% for *P*. *scapulatus*. The majority of PTTs had a duty cycle of 72 h off and 10 h on, providing multiple positional fixes every fourth day. Initial QLD deployments also trialed 48 h off, 10 h on, and 96 h off, 10 h on. The PTTs fitted to male *P*. *poliocephalus* in NSW had the longest duty cycle of 254 h off, 10 h on. A sparse duty cycle was chosen to maximize battery recharge and transmitter functionality based on the outcomes of previous studies [26, 85]. During on periods, the PTTs transmitted locational data to orbiting NOAA satellites, which relayed the data via ARGOS.

### Data handling and analysis

We analyzed all data in the R environment for statistical computing [86]. We managed data from deployed PTTs in a standardized format in Movebank (http://www.movebank.org/node/2). Prior to analysis, we examined the datasets for inconsistencies, and fixes with ARGOS code Z, along with fixes with longitudes > 140 or latitudes < 0, were removed. We used daytime fixes (between 10 am and 4 pm) to assign animals to a “roost site” (as mainland Australian flying-foxes do not forage during the day). If high resolution (ARGOS location code 3) daytime fixes occurred within 3.5 km of a “known colony” [66, 87], we assumed animals were roosting at that site. Where accurate daytime fixes were more than 3.5 km from a known roost location, we manually assigned animals to a new “roost site” located at the center of the cluster of fixes. If multiple tracked individuals roosted at the same location, this new roost site was confidently considered to be a previously unidentified “colony” of flying-foxes.

### Jurisdictions

There are three levels of government in Australia: local, state, and federal, each with their own elected decision-making bodies and responsibilities [88], and each with different implications for flying-fox management (see Discussion). The local level of government is usually called the city council or shire council (local council) headed by a Mayor or Shire President. The state level of government is subdivided in “state electorates” with elected representatives known as “Members” of the Legislative Assembly; the federal level of government is subdivided in “federal electorates” with elected representatives known as “Members” of the House of Representatives. To examine the movements of tracked flying-foxes among local councils, and state and federal electorates, we used roost locations to extract jurisdictional boundary data from shapefiles representing local government areas (LGAs), and state and federal electorates, using the R package “sp” [89]. Shapefiles were downloaded from the Australian Bureau of Statistics website (https://data.gov.au/dataset/ds-dga-bdcf5b09-89bc-47ec-9281-6b8e9ee147aa/details?q).

### Movements between roost sites

To test whether there were differences in roost site fidelity (i.e., the inverse of the probability of moving between roosts) between species, we constructed candidate generalized linear mixed effects models [90], including individual identity as a random effect. The global model had a binary response variable of 1 if an animal switched roosts between successive positional fixes and included the interaction between species and time between daytime fixes (in days) as explanatory variables. The variation in time between fixes was caused by differences in duty cycle, missed fixes, or a lack of positional fixes during daylight hours. The best fitting model was selected on the basis of AICc [91].

### Distance moved between roost sites

To test whether there were differences in the distance moved between roosts for the different species, we constructed candidate linear mixed effects models [90] with individual identity as a random factor. The global model had the natural log of the distance between fixes as the response variable and the interaction between species and the natural log of time (in days) between daytime fixes as explanatory variables. The best fitting model was selected on the basis of AICc and included a significant interaction between species and time between daytime fixes (days) (Additional file 11: Table S3). We took the coefficient “*p*” from the best fitting model for each species separately and used this to estimate the constant “*a*” to model the distance moved between daytime fixes using a power function [*f*(*x*) = *ax*^*p*^]. This was necessary as when time between successive daytime fixes was longer, it was more likely that roost locations were missed and therefore that the observed straight-line distance between fixes was shorter than the actual straight-line distance moved between roost locations. We used this to model the expected average distance between roosts that individuals from each species would be likely to move in a single day.

### Directional movements

To test whether animals coincided in the direction of their movement on a monthly basis, the bearing between each individual’s first and last monthly location was determined. These monthly bearings were plotted for each species separately. These data were used to examine whether they fell into one or more “preferred directions” using the Hermans-Rasson test [92]. The Bonferroni correction was used to account for the number of individual tests performed (i.e., by dividing the standard 0.05 significance level α by the number of tests performed for each species [93]). In months when a departure from uniformity was detected by the Hermans-Rasson test, a Rayleigh test [94] was also applied to examine whether the departure from uniformity consisted of a single peak, i.e., whether individuals of each species were significantly oriented (in the same direction) each month.

To test whether the three species performed seasonal north-south movements, a daily mean latitude (relative to capture location) was calculated, and a rolling average was calculated over a 30-day window for each species separately. Where animals were tracked for multiple years (*P*. *alecto* and *P*. *poliocephalus*), we calculated the mean monthly relative latitude of roosting locations and used the “ets” function of the R package forecast [95] to test whether seasonality was present in the dataset.

## Supplementary information

**Additional file 1: Table S1.** Details of study subjects.

**Additional file 6: Table S2.** Candidate generalized linear mixed effects models explaining the probabilities of switching roosts between successive daytime fixes.

**Additional file 7: Figure S1.** The relationships between the number of days between fixes and distance traveled between roosts, for the three different species. Black dots represent the means for each time step (days), ± 1 SE and lines are generated from the power function.

**Additional file 8: Figure S2.** The relationships between the cumulative distance traveled between roosts and the total number of days over which individuals were tracked, for each species.

**Additional file 9: Figure S3.** Rose diagrams showing the direction animals moved between roosts each month for *P*. *alecto*, *P*. *poliocephalus* and *P*. *scapulatus* separately. The species is indicated at the top of each panel. Each plot is labeled for year and month (YYMM). YYMM labels printed in red and marked with an asterisk indicate that the movement directions are aggregated into one or more preferred directions (Hermans-Rasson test). Plots that also include red arrows are those where a Raleigh test indicates a single preferred direction. Red arrows indicate the mean direction, and length of arrows the extent to which the individuals coincided in direction of movement. An arrow of length 1 (radius of plot circle = 1) indicates all individuals that moved, moved in the same direction. “*n*” is the number of individuals of each species tracked each month. “prop” is the proportion of tracked individuals that moved. Black dots indicate the direction in which individuals moved. Rose diagrams shaded in gray indicate that < 6 individuals moved in a given month and therefore the data were not statistically analyzed.

**Additional file 10: Figure S4.** Annual patterns of latitudinal displacement of satellite-tracked flying-foxes relative to their location of capture. The mean latitudinal movement, calculated per day over a 5-day moving window, is shown by thick colored lines. Black indicates movement patterns of *P*. *alecto*, blue indicates movements of *P*. *poliocephalus* and red indicates movements of *P*. *scapulatus*. Gray polygons represent 50 and 95% confidence intervals. The horizontal dashed line indicates no relative latitudinal displacement.

**Additional file 11: Table S3.** Candidate linear mixed effects models explaining the distance traveled between successive daytime fixes.

## Data Availability

The datasets analyzed during the current study are available in the Dryad database; 10.5061/dryad.mcvdncjz2 [96].
