# Supplementary material for: Extreme mobility of the world’s largest flying mammals creates key challenges for management and conservation
Source: BMC Biol. 2020 Aug 21;18:101. doi: 10.1186/s12915-020-00829-w (PMC7440933; doi:10.1186/s12915-020-00829-w)
Supplement: Supplementary file 5 — Additional file 1: Table S1. Details of study subjects. [file 12915_2020_829_MOESM1_ESM.docx]

| **Supplementary Table 1.** Details of study subjects | | | |  |
| --- | --- | --- | --- | --- |
|  | **N** | **Body mass (g)**  Mean (min, max) | **Number of days transmitting**  Mean (min - max) | |
| ***P. alecto*** | | | | |
| Female | 47 | 698 (516, 885) | 105 (23-367) | |
| Male | 33 | 785 (456, 903) | 113 (10-425) | |
| ***P. poliocephalus*** | | | | |
| Female | 51 | 753 (600, 920) | 746 (66-1809) | |
| Male | 58 | 820 (668, 990) | 272 (51-666) | |
| ***P. scapulatus*** | | | | |
| Female | 5 | 427 (345, 504) | 126 (15-194) | |
| Male | 7 | 472 (393, 504) | 95 (54-185) | |
