# Supplementary material for: Extreme mobility of the world’s largest flying mammals creates key challenges for management and conservation
Source: BMC Biol. 2020 Aug 21;18:101. doi: 10.1186/s12915-020-00829-w (PMC7440933; doi:10.1186/s12915-020-00829-w)
Supplement: Supplementary file 6 — Additional file 6: Table S2. Candidate generalized linear mixed effects models explaining the probabilities of switching roosts between successive daytime fixes. [file 12915_2020_829_MOESM6_ESM.docx]

**Supplementary Table 2.** Candidate generalized linear mixed effects models explaining the probabilities of switching roosts between successive daytime fixes.

| **Model^a^** | **AICc** | **ΔAICc** | **Weight** | **log(L)** | **df** |
| --- | --- | --- | --- | --- | --- |
| Species + Days | 12897.9 | 0.0 | 0.60 | -6444.0 | 5 |
| Species x Days | 12898.7 | 0.8 | 0.40 | -6442.4 | 7 |
| Days | 12918.7 | 20.8 | 0.00 | -6456.4 | 3 |
| Species | 13128.5 | 230.6 | 0.00 | -6560.3 | 4 |
| Null | 13157.6 | 259.7 | 0.00 | -6576.8 | 2 |

**^a^** Models are ranked by Akaike information criterion corrected to effective sample size (AICc) values calculated using the R package ‘MuMIn’. Change in AICc (ΔAICc), relative model weight (‘Weight’), log likelihood [log (L)] and degrees of freedom (df) are also included. Models include 11902 data points from 201 individuals.
