# Supplementary figures and images for: Extreme mobility of the world’s largest flying mammals creates key challenges for management and conservation
Source: BMC Biol. 2020 Aug 21;18:101. doi: 10.1186/s12915-020-00829-w (PMC7440933; doi:10.1186/s12915-020-00829-w)

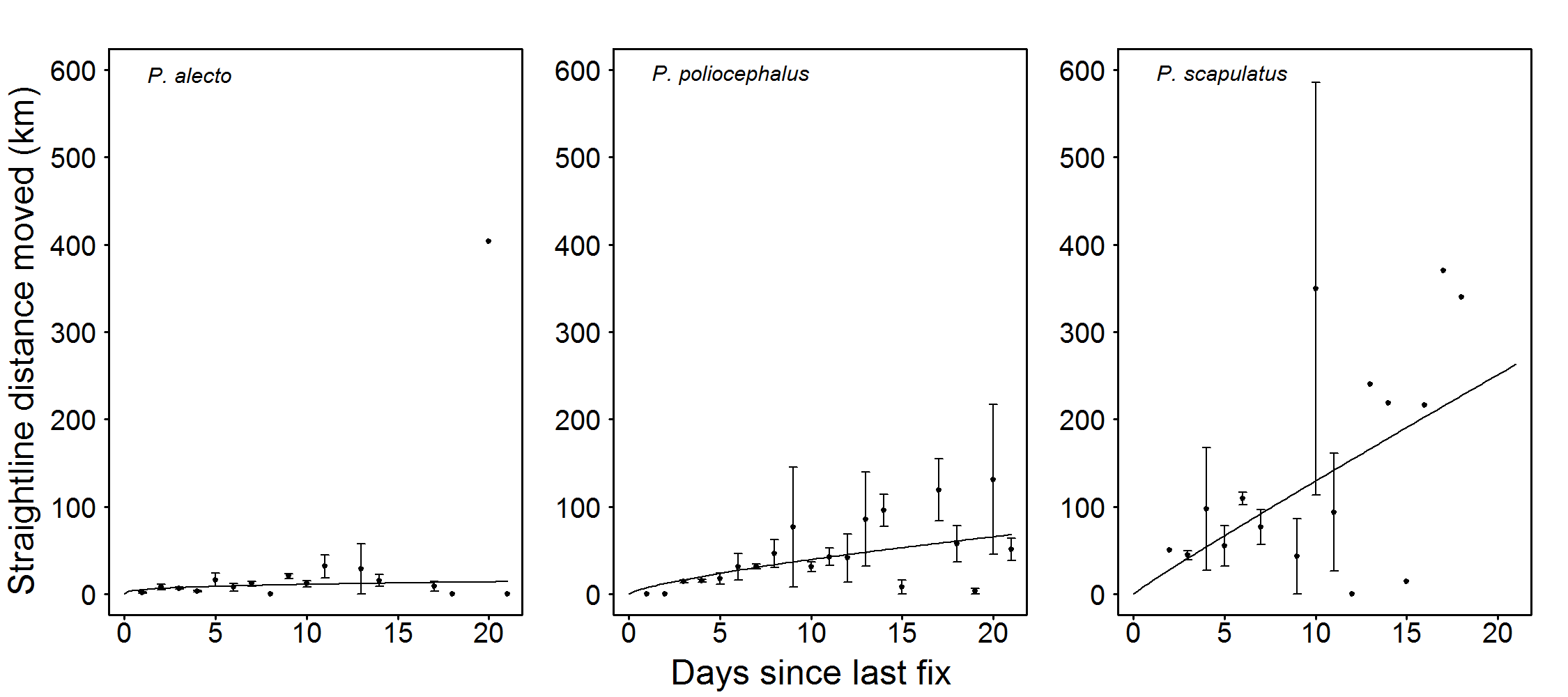

Supplement: Supplementary file 7 — Additional file 7: Figure S1. The relationships between the number of days between fixes and distance traveled between roosts, for the three different species. Black dots represent the means for each time step (days), ± 1 SE and lines are generated from the power function. [file 12915_2020_829_MOESM7_ESM.tiff]

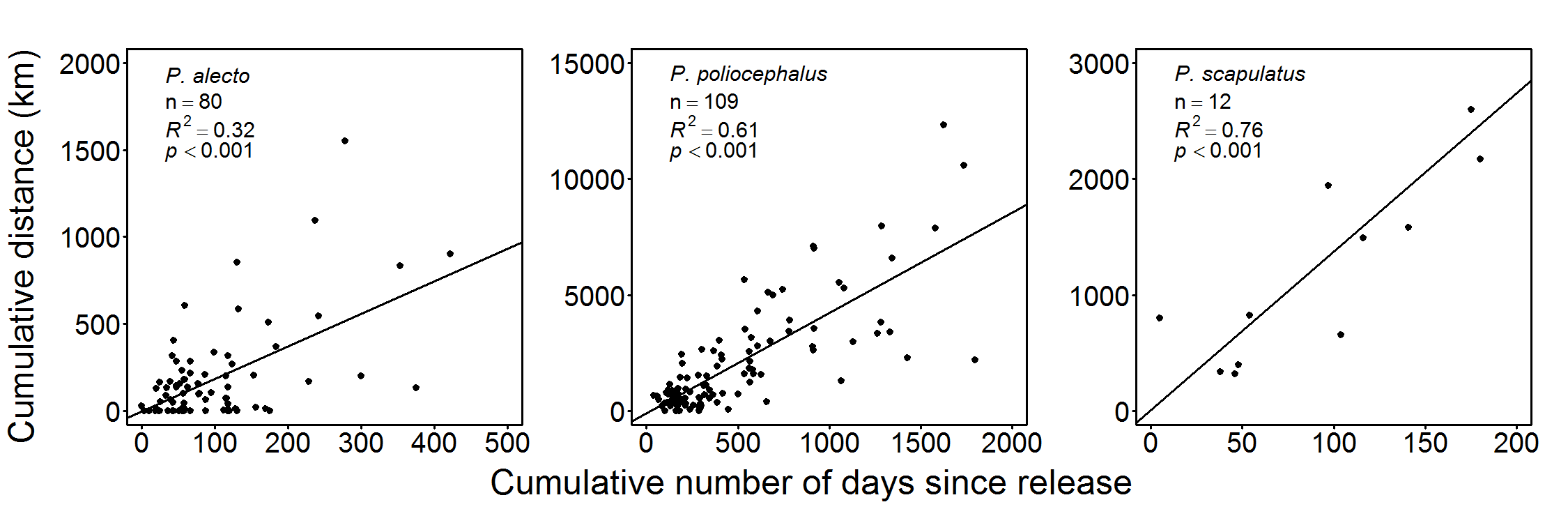

Supplement: Supplementary file 8 — Additional file 8: Figure S2. The relationships between the cumulative distance traveled between roosts and the total number of days over which individuals were tracked, for each species. [file 12915_2020_829_MOESM8_ESM.tiff]

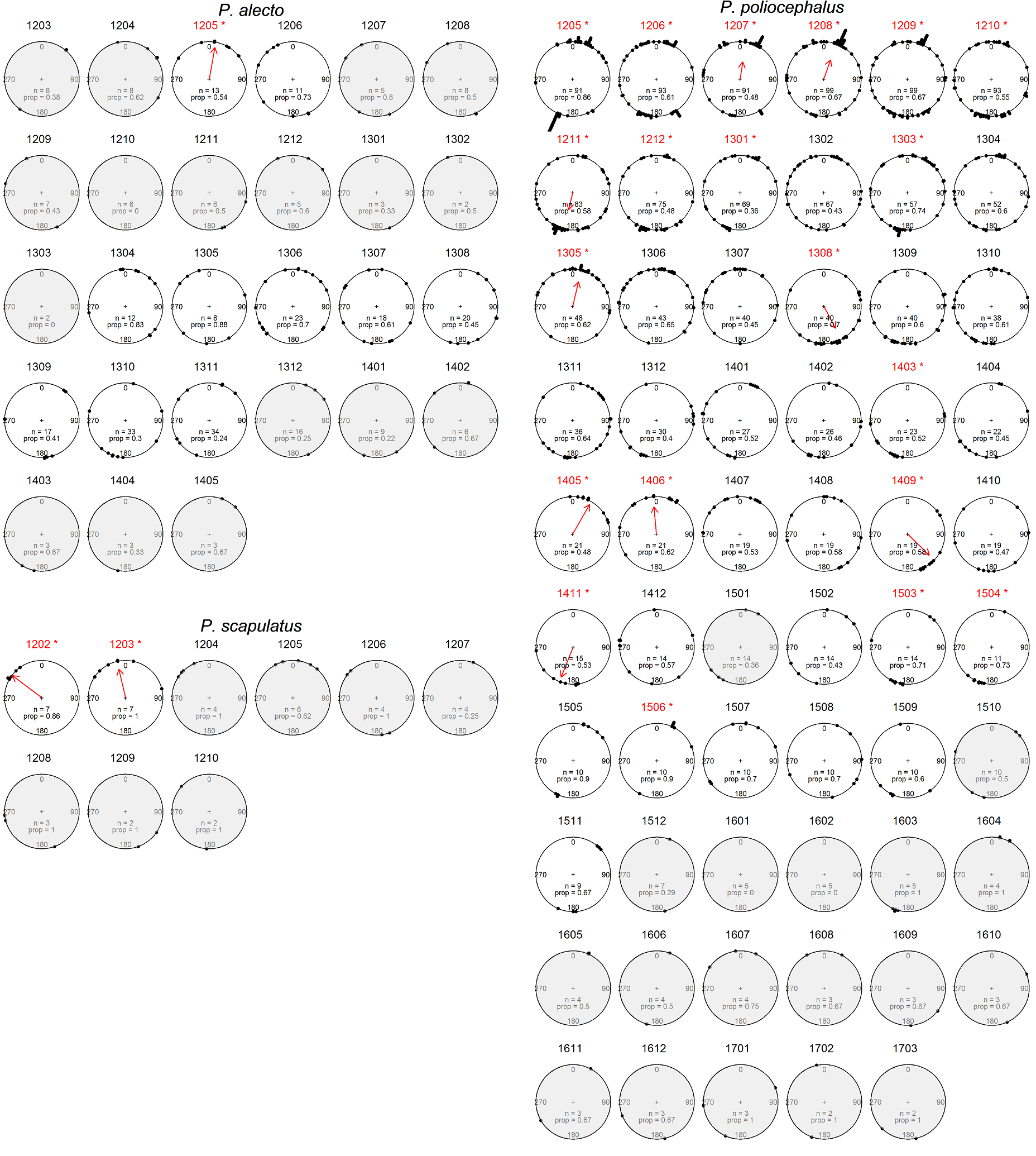

Supplement: Supplementary file 9 — Additional file 9: Figure S3. Rose diagrams showing the direction animals moved between roosts each month for P. alecto, P. poliocephalus and P. scapulatus separately. The species is indicated at the top of each panel. Each plot is labeled for year and month (YYMM). YYMM labels printed in red and marked with an asterisk indicate that the movement directions are aggregated into one or more preferred directions (Hermans-Rasson test). Plots that also include red arrows are those where a Raleigh test indicates a single preferred direction. Red arrows indicate the mean direction, and length of arrows the extent to which the individuals coincided in direction of movement. An arrow of length 1 (radius of plot circle = 1) indicates all individuals that moved, moved in the same direction. “n” is the number of individuals of each species tracked each month. “prop” is the proportion of tracked individuals that moved. Black dots indicate the direction in which individuals moved. Rose diagrams shaded in gray indicate that < 6 individuals moved in a given month and therefore the data were not statistically analyzed. [file 12915_2020_829_MOESM9_ESM.tif]

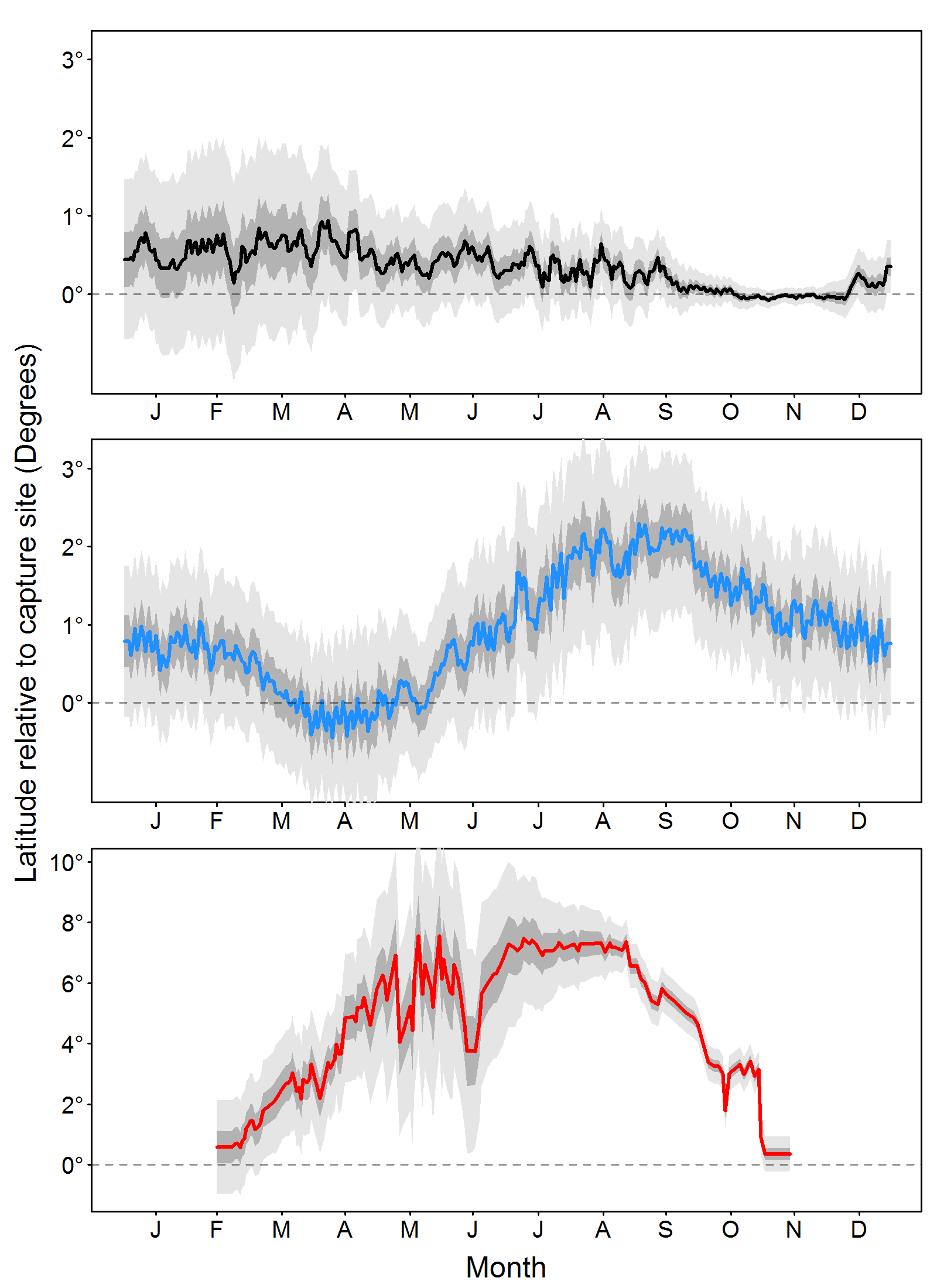

Supplement: Supplementary file 10 — Additional file 10: Figure S4. Annual patterns of latitudinal displacement of satellite-tracked flying-foxes relative to their location of capture. The mean latitudinal movement, calculated per day over a 5-day moving window, is shown by thick colored lines. Black indicates movement patterns of P. alecto, blue indicates movements of P. poliocephalus and red indicates movements of P. scapulatus. Gray polygons represent 50 and 95% confidence intervals. The horizontal dashed line indicates no relative latitudinal displacement. [file 12915_2020_829_MOESM10_ESM.tiff]
