# Supplementary material for: Extreme mobility of the world’s largest flying mammals creates key challenges for management and conservation
Source: BMC Biol. 2020 Aug 21;18:101. doi: 10.1186/s12915-020-00829-w (PMC7440933; doi:10.1186/s12915-020-00829-w)
Supplement: Supplementary file 11 — Additional file 11: Table S3. Candidate linear mixed effects models explaining the distance traveled between successive daytime fixes. [file 12915_2020_829_MOESM11_ESM.docx]

**Supplementary Table 3.** Candidate linear mixed effects models explaining the distance travelled between successive daytime fixes.

| **Model^a^** | **AICc** | **ΔAICc** | **Weight** | **log(L)** | **df** |
| --- | --- | --- | --- | --- | --- |
| Species x Days | 45062.4 | 0.0 | 0.97 | -22523.2 | 8 |
| Species + Days | 45069.4 | 7.0 | 0.03 | -22528.7 | 6 |
| Days | 45154.0 | 91.6 | 0.00 | -22574.0 | 3 |
| Species | 45353.6 | 291.2 | 0.00 | -22671.8 | 5 |
| Null | 45385.6 | 323.2 | 0.00 | -22689.8 | 3 |

**^a^** Models are ranked by Akaike information criterion corrected to effective sample size (AICc) values calculated using the R package ‘MuMIn’. Change in AICc (ΔAICc), relative model weight (‘Weight’), log likelihood [log (L)] and degrees of freedom (df) are also included. The natural log of both distance travelled and days between fixes were taken. Models include 11902 data points from 201 individuals.
